# The Book World of Medicine and Science

**Published:** 1895-07-13

**Authors:** 


### The Book World of Medicine and Science.

Diseases of the Nose and Throat. By F. De Haviland Hall, M.D., F.R.C.P. Practical Series. (London: H. K. Lewis. 1894.)

There can be no doubt that this work well deserves a place amongst a practical series. From a casual perusal of the preface the reader is rather prepared for a theoretical and abstract treatise dealing with the most recent and up-to-date laryngology and rhinology. This is far from being a correct view, since from start to finish Dr. De Haviland Hall's work is full of information and suggestions of the most practical nature. There is an agreeable avoidance of controversial disagreement as regards pathology and symptomatology, and a most refreshing presence of hard and fast rules for treatment, instead of, as is so often the case, a number of alternative methods, which leave the inexperienced in hopeless doubt as to the best to select. Dr. Hall's statements are commendably free from prejudice and extreme views. Rhinologists have too often made themselves ridiculous by attributing to the special diseases of their branch of medicine all the neurotic symptoms which the general physician would rather regard as belonging to his own particular department. Dr. Hall is careful to avoid all cause for inclusion amongst this class of specialists. Speaking of deviations, spurs, and crests of the nasal septum, the author remarks: "Judging from the writings of some rhinologists it would seem that in the nose we would have the key of the most complex processes, and that the rectification of septal abnormalities would suffice to cure symptoms referred to the most distant organs. Now, however, that the first burst of enthusiasm is over, there is every reason to believe that the opinion of the moderate men will prevail, and that deformities of the septum will not be interfered with unless there are clear grounds for supposing that surgical interference is absolutely necessary." This passage is thoroughly typical of the whole tenor of the book, and demonstrates the fair-mindedness and even balance of the author's views. The article on diphtheria was probably written before the introduction of the antitoxin treatment which probably accounts for the omission of this method of treatment.

Practitioners and students will, when familiar with this new treatise, doubtless agree in regarding as unnecessary the author's apology for "adding another book to the long list of those dealing with diseases of the nose and throat." It is doubtless the most practical book on these subjects, and practitioners who refer to it will be saved from many a difficulty, and often find a ready solution of those nose and throat problems which daily perplex the inexperienced. Many of the illustrations are new and original, and those devoted to the section on laryngoscopy are of special value, and include two excellent coloured plates.

